# Calotropin and corotoxigenin 3-O-glucopyranoside from the desert milkweed *Asclepias subulata* inhibit the Na^+^/K^+^-ATPase activity

**DOI:** 10.7717/peerj.13524

**Published:** 2022-06-02

**Authors:** Salvador E. Meneses-Sagrero, Luisa A. Rascón-Valenzuela, Juan C. García-Ramos, Wagner Vilegas, Aldo A. Arvizu-Flores, Rogerio R. Sotelo-Mundo, Ramon E. Robles-Zepeda

**Affiliations:** 1 Ciencias Químico Biológicas, Universidad de Sonora, Hermosillo, Sonora, México; 2 Escuela de Ciencias de la Salud, Universidad Autónoma de Baja California, Ensenada, Baja California, México; 3 Instituto de Biociências, São Paulo State University, Sao Paulo, Brasil; 4 Laboratorio de Estructura Molecular, Centro de Investigación en Alimentación y Desarrollo AC, Hermosillo, Sonora, México

**Keywords:** Cardenolides, ATPase activity, *Asclepias subulata*, Uncompetitive inhibition

## Abstract

Na^+^/K^+^-ATPase is an essential transmembrane enzyme found in all mammalian cells with critical functions for cell ion homeostasis. The inhibition of this enzyme by several cardiotonic steroids (CTS) has been associated with the cytotoxic effect on cancer cell lines of phytochemicals such as ouabain and digitoxin. This study evaluated the inhibitory capacity of cardenolides calotropin and corotoxigenin 3-O-glucopyranoside (C3OG) from *Asclepias subulata* over the Na^+^/K^+^-ATPase activity *in vitro* and *silico*. The inhibitory assays showed that calotropin and C3OG decreased the Na^+^/K^+^-ATPase activity with IC_50_ values of 0.27 and 0.87 μM, respectively. Furthermore, the molecules presented an uncompetitive inhibition on Na^+^/K^+^-ATPase activity, with K_i_ values of 0.2 μM to calotropin and 0.5 μM to C3OG. Furthermore, the molecular modeling indicated that calotropin and C3OG might interact with the Thr^797^ and Gln^111^ residues, considered essential to the interaction with the Na^+^/K^+^-ATPase. Besides, these cardenolides can interact with amino acid residues such as Phe^783^, Leu^125^, and Ala^323^, to establish hydrophobic interactions on the binding site. Considering the results, these provide novel evidence about the mechanism of action of cardenolides from *A. subulata*, proposing that C3OG is a novel cardenolide that deserves further consideration for *in vitro* cellular antiproliferative assays and *in vivo* studies as an anticancer molecule.

## Introduction

Phytochemicals are potent molecules isolated from plants with powerful beneficial functions, such as antineoplastic (Patra et al., 2021). Cancer is a complex multifactorial disease involving mutations, carcinogens, and environmental factors that remain elusive towards an early diagnostic and personalized treatment (Kandoth et al., 2013; Smith, 2013). Recently, efforts have been focused on developing therapies with high selectivity for tumor tissue to reduce side effects (Choudhari et al., 2020). The sodium-potassium ATPase has been a potential and attractive target for cancer therapy in the past few years for its role in signal transduction, ion homeostasis, and overexpression in severe neoplasias (Bejček et al., 2021). The enzyme Na^+^/K^+^-ATPase is a selective channel in all mammalian cells’ plasma membrane that responds to sodium and potassium ion gradients and regulates extracellular signals and the membrane potential (Clausen, Hilbers & Poulsen, 2017). This enzyme is a glycoprotein composed of two major subunits, a catalytic α-subunit involved in hydrolyzing ATP and the β-subunit. Some cells express a third regulatory subunit, the γ-subunit (Pu, Scanzano & Blostein, 2002). Multiples isoforms exist for α (α1–4) and β (β1–3) subunits, which are tissue-specific and developmentally regulated expression patterns (Habiba, Blanco & Mercer, 2000; Horvat et al., 2006). As for the transport activity, the protein exports three Na^+^ ions in exchange for two K^+^ ions imported into the cell. This function requires ATP and Mg^2+^, and the ligand-binding step includes phosphorylation, accompanied by conformational changes associated with the ion transport (Pivovarov, Calahorro & Walker, 2019). The Na^+^/K^+^-ATPase is sensible to cardiotonic steroids (CTS) (Lingrel, 2010).

The fact that CTS target the Na^+^/K^+^-ATPase and leads to a sodium/potassium unbalance in the cell is one of the reasons for the great interest in these phytochemical compounds (Prassas & Diamandis, 2008; Yatime et al., 2011).

In cardiac cells, the inhibition of Na^+^/K^+^-ATPase by CTS molecules promotes the influx of intracellular Na^+^ and the decrease of K^+^ in myocytes. The intracellular Na^+^ modulates the activity of the Na^+^/Ca^2+^ exchanger at the membrane, inducing an increase in the levels of Ca^2+^ in the cell that promote an increase in the cardiac tissue contractile force. Besides, the rise in intracellular Ca^2+^ levels provoked by the CTS over the Na^+^/K^+^-ATPase leads to an increase in the cardiac muscle tone and circulating blood volume and a decrease in the heart rate and stroke volume (Zanchett-Schneider et al., 2017).

**Figure 1:**
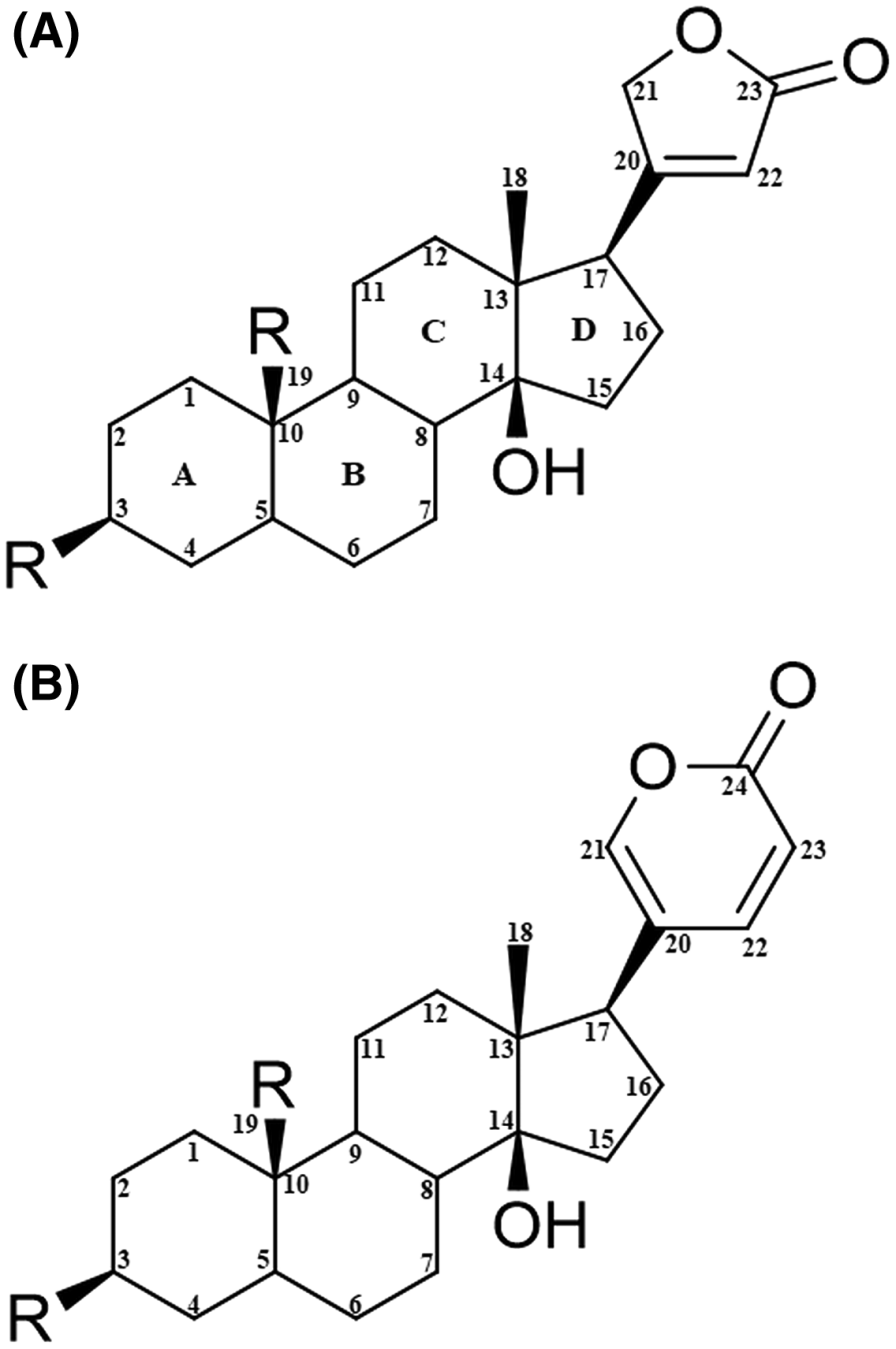
Structural nomenclature of cardiac glycosides. (A) Cardenolides. (B) Bufadienolides.

The CTS molecule has a steroidal core with four rings (A, B, C, and D) and one or more sugar moieties on the ring at position 3 (Fig. 1). They are classified into two subgroups based on the substitution at position 17: cardenolides, if they present a butyrolactone ring; or bufadienolides, if they present an α-pyrone ring (Diederich, Muller & Cerella, 2017). In recent years, cardenolides have emerged as a possible alternative to anticancer therapeutics due to their biological activity of binding to Na^+^/K^+^-ATPase (Škubnik et al., 2021).

Cardiac glycosides can activate intracellular signaling pathways to a certain degree due to their association with Na^+^/K^+^-ATPase, which leads to protein kinases activation (Banerjee et al., 2018). However, these signal pathways depend on the type of cardenolide and the nature of cancer cells. Therefore, it is essential to acknowledge that cardenolides can activate more than one signaling pathway.

Cardenolides as ouabain can activate intracellular pathways such as Src/EGFR-Ras-Raf-ERK1/2 and PI3K-PDK-Akt, activate protein/lipid kinase cascades, generate ROS and stimulate Ca^2+^ oscillation by a cell specific-manner, triggering cell death, survival or proliferation (Cui & Xie, 2017). Other cardenolides such as oleandrin may trigger mechanisms that involve the alteration of membrane fluidity and the decrease of activation of nuclear transcription factors such as NF-κB, JNK, and AP-1, causing oxidative and mitochondrial injury (Wen et al., 2016). Additionally, a semisynthetic cardenolide UNBS1450 inhibits oncogene cMyc, associated with cell migration through the Wnt/β-catenin pathway (Silva et al., 2021), and molecules as proscillaridin A can inhibit topoisomerase I (Durlancher et al., 2015).

Recently, phytochemicals have received significant attention as anticancer agents due to their capacity to induce apoptosis (Cerella, Dicato & Diederich, 2013). *Asclepias* is a genus of perennial plants widely distributed in North America, with 164 species divided into nine subgenera. Mexico has around 68 *Asclepias* species, 14 of which have medicinal properties. *Asclepias subulata* is a shrub from the west of Arizona in the United States and Baja California and Sonora in the Northwest of Mexico (Fernandez-Brewer, Juárez-Jaimes & Cortes-Zárraga, 2008). Different plant parts are used by Sonoran ethnic groups, such as Seris or Pimas, to treat health problems such as gastrointestinal disorders, eye disease, and cancer, among others (Johnson-Gordon, Moreno-Salazar & López-Estudillo, 1996). The phytochemical analysis of *Asclepias* extracts revealed the presence of lignans, terpenoid compounds, alkaloids indole-type, and some cardenolides like uscharin, calactin, frugoside, and calotropin (Jolad et al., 1986). More recently, other novel compounds were isolated and identified from a methanolic extract of *A. subulata*, such as calotropin, 12, 16-dihydroxycalotropin, corotoxigenin 3-O-glucopyranoside (C3OG), and desglucouzarin.

These compounds were evaluated against a panel of human cancer cell lines (A549, PC-3, LS-180), the non-cancerous human cell line (ARPE-19), and a murine cancer cell line (RAW 264.7), where the compounds had IC_50_ values in the nM range against human tumoral cancer cell lines (Rascón-Valenzuela et al., 2015). They also showed selective cytotoxicity by not significantly affecting the non-cancerous human cell line and the murine cancer cell line (Rascón-Valenzuela et al., 2015). Additionally, these cardenolides induced cell death through caspase-dependent apoptosis, activated by an extrinsic pathway (Rascón-Valenzuela et al., 2016). Furthermore, recent studies have shown that calotropin inhibits the enzymatic activity of the Na^+^/K^+^-ATPase (Pederson et al., 2020; Agrawal et al., 2021). Therefore, this study aimed to accomplish enzymatic inhibition assays *in vitro* to determine the effect of calotropin and C3OG on the Na^+^/K^+^-ATPase activity and perform the molecular docking of these cardenolides as ligands with a structural Na^+^/K^+^-ATPase model, to propose the possible conformation and interaction of the molecules.

## Materials and Methods

### Material and reagents

Brain porcine adenosine 5′-triphosphatase (Na^+^/K^+^-ATPase), adenosine 5′-triphosphate (ATP), ouabain octahydrate, Trizma® base, magnesium chloride (MgCl_2_), sodium chloride (NaCl), potassium chloride (KCl), dimethyl sulfoxide (DMSO), trichloroacetic acid (TCA), iron (II) sulfate heptahydrate, ammonium molybdate, sulfuric acid, ethylenediaminetetraacetic acid (EDTA), Bradford reagent, and bovine serum albumin (BSA) were purchased from Sigma-Aldrich Chemical Co. (St. Louis, MO, USA). All reagents and solutions used in the assays were prepared with Milli-Q® water (Merck, Darmstadt, Germany). The compounds calotropin and C3OG were previously isolated and characterized from *A. subulata* in our working group (Rascón-Valenzuela et al., 2015).

### *In vitro* inhibitory assay of Na^+^/K^+^-ATPase

We used the inhibitory assay method described by Mugpasao et al. (2014). The enzymatic activity of Na^+^/K^+^-ATPase was determined by colorimetric quantification of orthophosphate (Pi) released during the ATP hydrolysis according to the method of Taussky, Shorr & Kurzmann (1953). The inhibition assays were done using commercial brain porcine Na^+^/K^+^-ATPase. Protein concentration was determined according to the Bradford method (Bradford, 1976) using bovine serum albumin as standard. As inhibitors, we used cardenolides ouabain, calotropin, and C3OG. The cardenolide ouabain was used as a reference molecule. The reaction was done in a 96-wells plate, adding 10 μL of Na^+^/K^+^-ATPase solution (0.5 U/mL) with 4 μL of NaCl/KCl solution (35 mM of KCl and 1.65 M of NaCl), 70 μL of Tris-HCl buffer (30 mM Tris-HCl, 0.5 mM of EDTA and 7.8 mM of MgCl_2_) at 37 °C and 30 min with 6 μL either dimethyl sulfoxide (DMSO) or test compounds on each well. The range concentrations of inhibitors were 100.0–0.001 μM of ouabain and 1000.0–0.01 μM of calotropin and C3OG. Then, 20 μL of ATP solution 22 mM was added to each well, and the reaction solution was incubated at 37 °C for 15 min.

Next, we added 30 μL of trichloroacetic acid (TCA) at 30% (w/v) to each well to block the reaction. The 96-wells plate was mixed slightly and centrifuged at 3,000 rpm for 15 min. Finally, 50 μL of the supernatant was transferred to another 96-well plate and were coated with 100 μL of Taussky-Shorr solution. The absorbance was read at 660 nM in a microplate reader ThermoScientific Multiskan (Thermo Fisher, Vantaa, Finland) after incubation of 5 min at room temperature, protected from the light. The color intensity was proportional to the release of orthophosphate, indicating ATP hydrolysis and the Na^+^/K^+^-ATPase activity. The inhibition of the enzymatic activity by cardenolides was managed in the percentage of Na^+^/K^+^-ATPase activity compared with control. The data of inhibitory Na^+^/K^+^-ATPase activity were expressed in IC_50_ and standard deviation. All experiments were performed in triplicate.

### Kinetic analysis

The Michaelis-Menten kinetic parameters K_m_ and V_max_ of porcine brain Na^+^/K^+^-ATPase were determined from the initial velocities by modifying the ATP concentrations from 0.125 to 4.0 mM using the enzymatic standard conditions previously mentioned. Initial rates were recorded for 15 min in intervals of 3 min. The measurements were done in the absence or presence of inhibitors. The concentration of Na^+^, K^+^, and Mg^2+^ ions remained constant. The initial velocity when the *A. subulata* cardenolides were tested was recorded for 15 min. Kinetic analyses were carried out in the presence or absence of two different concentrations of cardenolides (0.1 and 1.0 μM) to determine the nature of the inhibition of the Na^+^/K^+^-ATPase. All experiments were performed in triplicate. The experimental data were fitted to the Michaelis-Menten equation by non-linear regression analysis. The type of inhibition was obtained by Lineweaver-Burk double reciprocal plots using GraphPad Prism software version 8 (GraphPad Software Inc., San Diego, CA, USA).

### Molecular modeling

#### Ligand and receptor preparation

**Figure 2:**
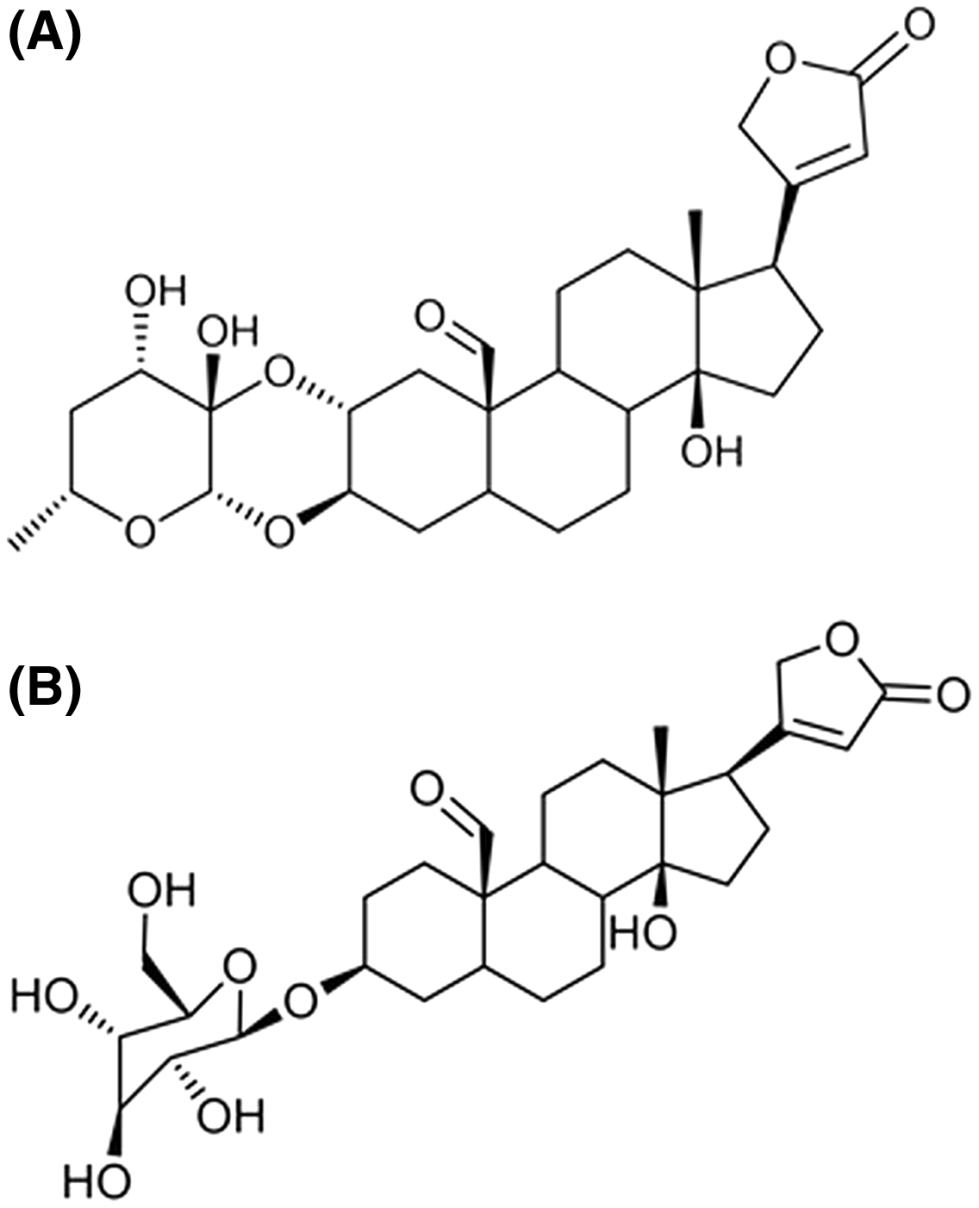
Structure of the cardenolides used for molecular modeling. (A) Calotropin. (B) Corotoxigenin 3-O-glucopyranoside.

Data files for the structure of the following cardenolides were prepared for docking: calotropin, corotoxigenin 3-O-glucopyranoside, and ouabain. First, ChemDraw Ultra V.12 software (PerkinElmer Inc., Waltham, MA, USA) was used to build the 2D structures for the docked ligands and then exported to Molecular Operating Environment (MOE) software 2018 (Chemical Computer Group ULC, Montréal, QC, Canada) to convert these into 3D models. Then, the MMFF94 forcefield was applied to structures for energy minimization to obtain a conformer library. The molecular structure of the ligands used in this work are shown in Fig. 2.

The porcine kidney Na^+^/K^+^-ATPase (PDB code: 4HYT) enzyme X-ray crystallographic structure was obtained from the Protein Data Bank (https://www.rcsb.org). The structure had a 3.4 Å resolution and shared a 98% homology with the human protein (Laursen et al., 2013). The protein was prepared for the docking study by eliminating beta and gamma subunits, water molecules, and ligands not involved in the binding site. In addition, to prepare the alpha subunit, ouabain and magnesium were conserved as reference ligands. The alpha subunit protein was then prepared using the MOE software with the CHARMM27 forcefield to obtain the minimum energy conformation of the receptor.

### Molecular docking

Molecular docking studies were performed using Na^+^/K^+^-ATPase co-crystallized with ouabain as a reference ligand. The dataset of cardenolides previously mentioned was used as ligands to predict the binding affinity with the receptor. A specific cavity for the interaction between the reference ligand was selected for the docking calculations. According to the binding protocol on the MOE software, the binding site on the Na^+^/K^+^-ATPase was kept rigid, while cardenolides were kept as flexible molecules. Three independent stochastic series were conducted to obtain the convergence of the best scoring position. The alpha-triangle placement protocol was applied, where 60,000 poses were considered for each conformer using the London dG scoring function in MOE. The 30 top high-scoring poses for each ligand were subjected to a structural refinement with the induced-fit protocol.

The ligand-receptor complexes obtained through docking procedures were selected based on the binding energy expressed in kcal/mol and root mean square deviation (RMSD) values. The analysis of the molecular modeling, the images, and the diagram of interactions between the selected ligand poses and the amino acids were performed in Discovery Studio software version 2019 (Biovia, Dassault Systemes Biovia Corp, San Diego, CA, USA).

### Statistical analysis

The Na^+^/K^+^-ATPase inhibition plots were fitted to a non-linear regression analysis on the data assuming the classical concentration-response curve model to estimate the inhibitory concentration at 50% (IC_50_) on GraphPad Prism software version 8 (GraphPad Software Inc., San Diego, CA, USA). In addition, the means difference of the results of Na^+^/K^+^-ATPase activity was analyzed by one-way analysis of variance (one-way ANOVA) followed by the Tukey test using IBM SPSS software statistics version 20 (IBM Inc., Hong Kong, China).

## Results

### Effect of cardenolides on the enzymatic activity of Na^+^/K^+^-ATPase *in vitro*

**Figure 3:**
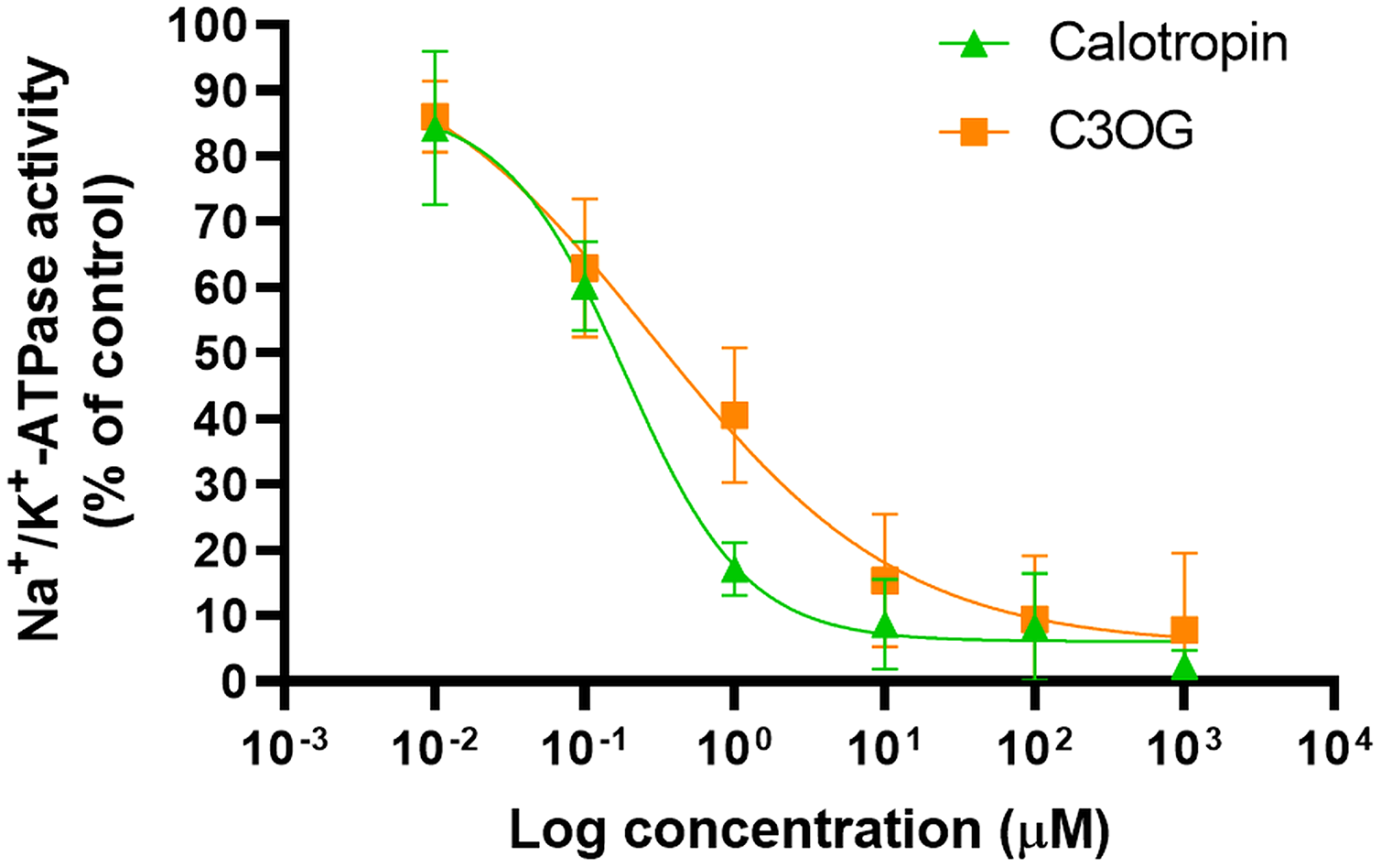
Inhibitory effect of cardenolides on brain porcine Na^+^/K^+^-ATPase enzymatic activity. Non-linear regression analysis of a concentration-response curve model estimates the inhibitory concentration at 50% (IC_50_). Green triangles depict results of calotropin and orange squares depict resullts of C3OG. The data come from three independent experiments performed in triplicate. The solid lines represent the fitted model.

We performed an inhibition assay to determine the inhibitory effect of calotropin and corotoxigenin 3-O-glucopyranoside (C3OG) on porcine brain Na^+^/K^+^-ATPase. The porcine brain Na^+^/K^+^-ATPase was treated with different concentrations of cardenolides. The Na^+^/K^+^-ATPase activity was 2.69 μmolPi/mg/min. Calotropin and C3OG presented a typical dose-response effect on the Na^+^/K^+^-ATPase activity (Fig. 3). This trend was similar to that observed in ouabain (Fig. S1). Also, as shown in (Table 1), calotropin presented a better inhibitory effect on Na^+^/K^+^-ATPase activity than C3OG, with IC_50_ values of 0.27 ± 0.06 μM and 0.87 ± 0.2 μM, respectively. Nevertheless, both cardenolides presented a lower IC_50_ value than ouabain, which showed an IC_50_ value of 0.12 ± 0.02 μM. The difference between the IC_50_ values among cardenolides suggests a contrast in how the compounds bind to the enzyme, probably due to the structural diversity of cardenolides isolated from *A. subulata* compared to ouabain.

**Table 1:** Inhibitory effect of cardenolides ouabain, calotropin, and corotoxigenin 3-O-glucopyranoside on the enzymatic activity of Na^+^/K^+^-ATPase.

Compound	Inhibition of Na^+^/K^+^-ATPase IC_50_ (μM)^a^ ± SD
Ouabain^b^	0.12 ± 0.02
Calotropin	0.27 ± 0.06
Corotoxigenin 3-O-glucopyranoside*	0.87 ± 0.2

**Notes:**

a Half maximal inhibitory. The results are expressed as IC_50_ ± standard deviation.

b Ouabain was used as a positive control of inhibition of Na^+^/K^+^-ATPase.

* Significative differences (*p* < 0.05) compared with the reference compound ouabain.

### Determination and kinetic parameters of cardenolides isolated from *A. subulata* on Na^+^/K^+^-ATPase

**Figure 4:**
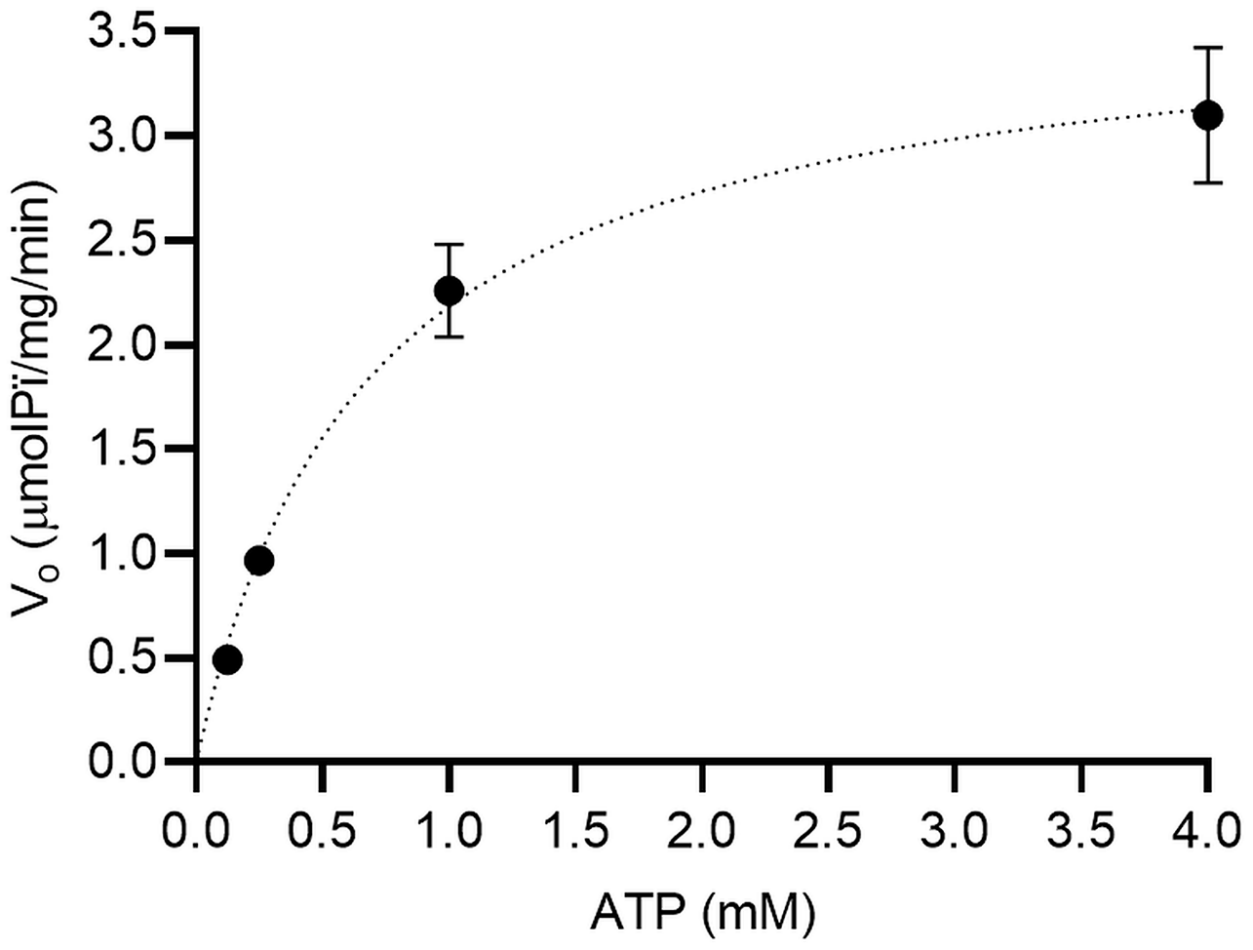
Michaelis-Menten kinetics for the substrate (ATP) on porcine brain Na^+^/K^+^-ATPase. The data were fitted to a Michaelis-Menten kinetics model using a non-linear regression model (R^2^ = 0.97). All substrate concentrations were evaluated by triplicate.

**Figure 5:**
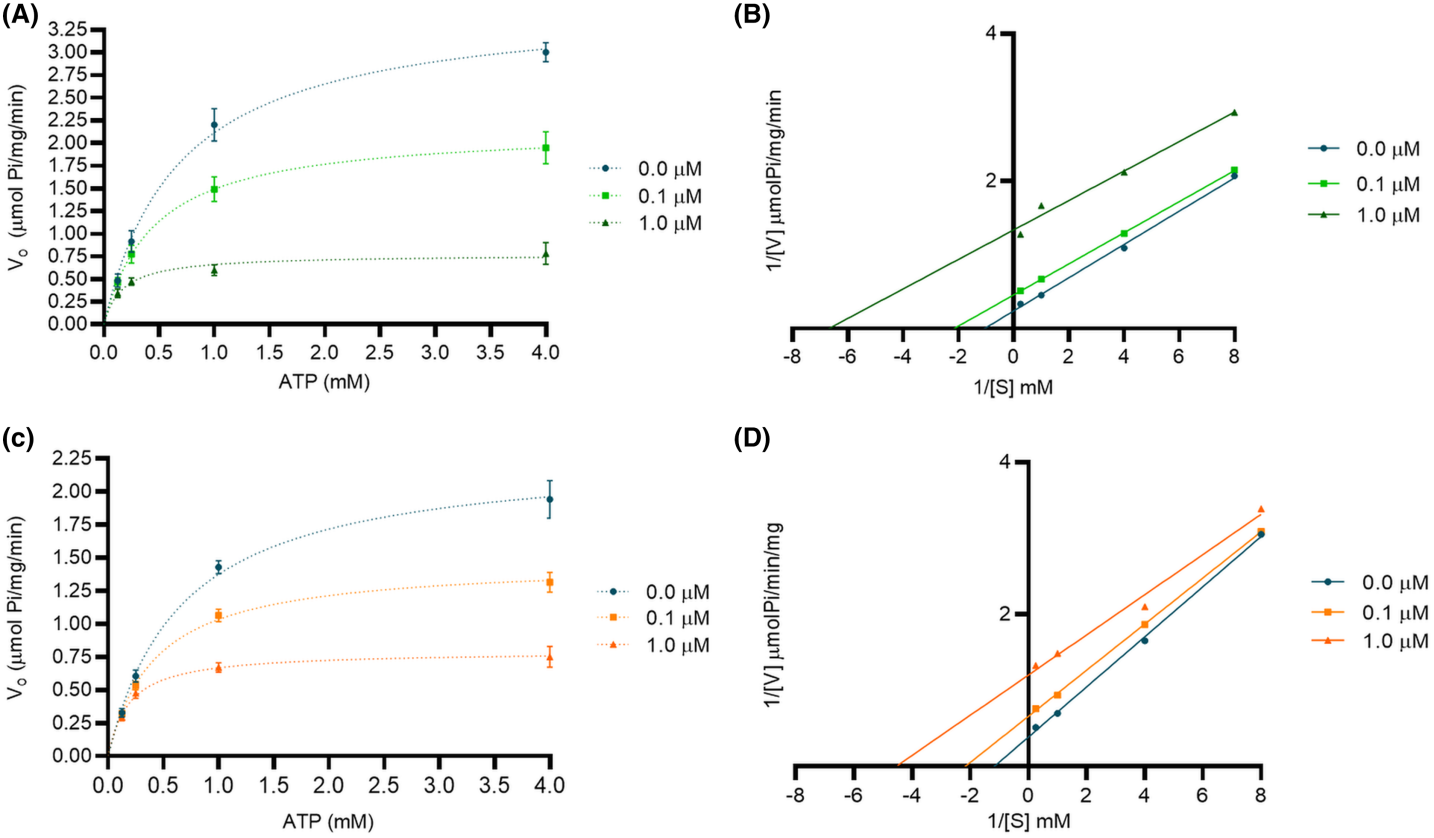
Mode of inhibition of cardenolides over Na^+^/K^+^-ATPase. (A) Michaelis-Menten modeling of calotropin inhibition at 0.1 μM (green squares), 1.0 μM (green triangles), and without inhibitor (blue circles). (B) Lineweaver-Burk plot of the data shown in panel A. (C) Michaelis-Menten modeling of C3OG inhibition at 0.1 μM (orange squares), 1.0 μM (orange triangles), and without inhibitor (blue circles). (D) Lineweaver-Burk plot of the data shown in panel C. Data were determined by three independent assays with three replicates.

We performed an enzymatic inhibition assay to confirm the calotropin and C3OG binding to Na^+^/K^+^-ATPase. We confirmed the Na^+^/K^+^-ATPase reaction occurs in classical Michaelis-Menten kinetics (Fig. 4). The apparent K_m_ and V_max_ values were 0.68 ± 0.08 mM and 3.6 ± 0.4 μmolPi/mg/min, respectively. Cardenolides from *A. subulata*, calotropin, and C3OG at 0.1 and 1.0 μM also presented the classical Michaelis-Menten kinetics (Figs. 5A and 5C). According to the apparent K_m_ and V_max_ values shown in (Table 2), calotropin and C3OG decreased the values of K_m_ and V_max_ of the Na^+^/K^+^-ATPase when the concentration of inhibitor increased, which indicates an uncompetitive inhibition type of the cardenolides on the Na^+^/K^+^-ATPase. The Lineweaver-Burk plot confirmed this for calotropin (Fig. 5B) and C3OG (Fig. 5D), which showed a parallel lines pattern at all inhibitor concentrations evaluated. This matches ouabain’s inhibition type in the Lineweaver-Burk plot (Fig. S2) and the K_m_ and V_max_ values (Table S1) as uncompetitive types. The K_i_ value determined the inhibition potency of the Na^+^/K^+^-ATPase activity by cardenolides. As presented in (Table S1), the Na^+^/K^+^-ATPase was inhibited by ouabain with a K_i_ value of 0.16 μM. The estimated K_i_ values for calotropin and C3OG were 0.20 and 0.52 μM, respectively (Table 2), suggesting a high affinity between the cardenolides and the Na^+^/K^+^-ATPase-substrate complex. The cardenolides in this study showed the same pattern in the IC_50_ and the K_i_ values, where ouabain had the most potent inhibition of the enzyme activity, followed by calotropin and C3OG.

**Table 2:** Kinetic analysis of Na^+^/K^+^-ATPase activity from the brain porcine in the absence or presence of cardenolides calotropin and C3OG at different concentrations.

Compound	Concentration (μM)	K_m_ (mM)	V_max_ (μmolPi/mg/min)	K_i_ (μM)	Type of inhibition
Calotropin	0.0	0.68 ± 0.12	3.56 ± 0.06	0.20 ± 0.03	Uncompetitive
	0.1	0.45 ± 0.07	2.17 ± 0.10		
	1.0	0.17 ± 0.03	0.77 ± 0.10		
C3OG^a^	0.0	0.66 ± 0.1	2.29 ± 0.10	0.52 ± 0.05	Uncompetitive
	0.1	0.42 ± 0.04	1.47 ± 0.08		
	1.0	0.12 ± 0.01	0.80 ± 0.06		

**Notes:**

a Corotoxigenin 3-O-glucopyranoside.

All values were obtained from triplicate independent assays. The data are expressed as mean ± standard deviation. The values of K_m_ and V_max_ were calculated by data fitting Michaelis-Menten non-linear regression. K_m_ is expressed in mM. V_max_ is expressed as μmol of Pi released/mg of protein/min.

### Molecular docking validation

**Figure 6:**
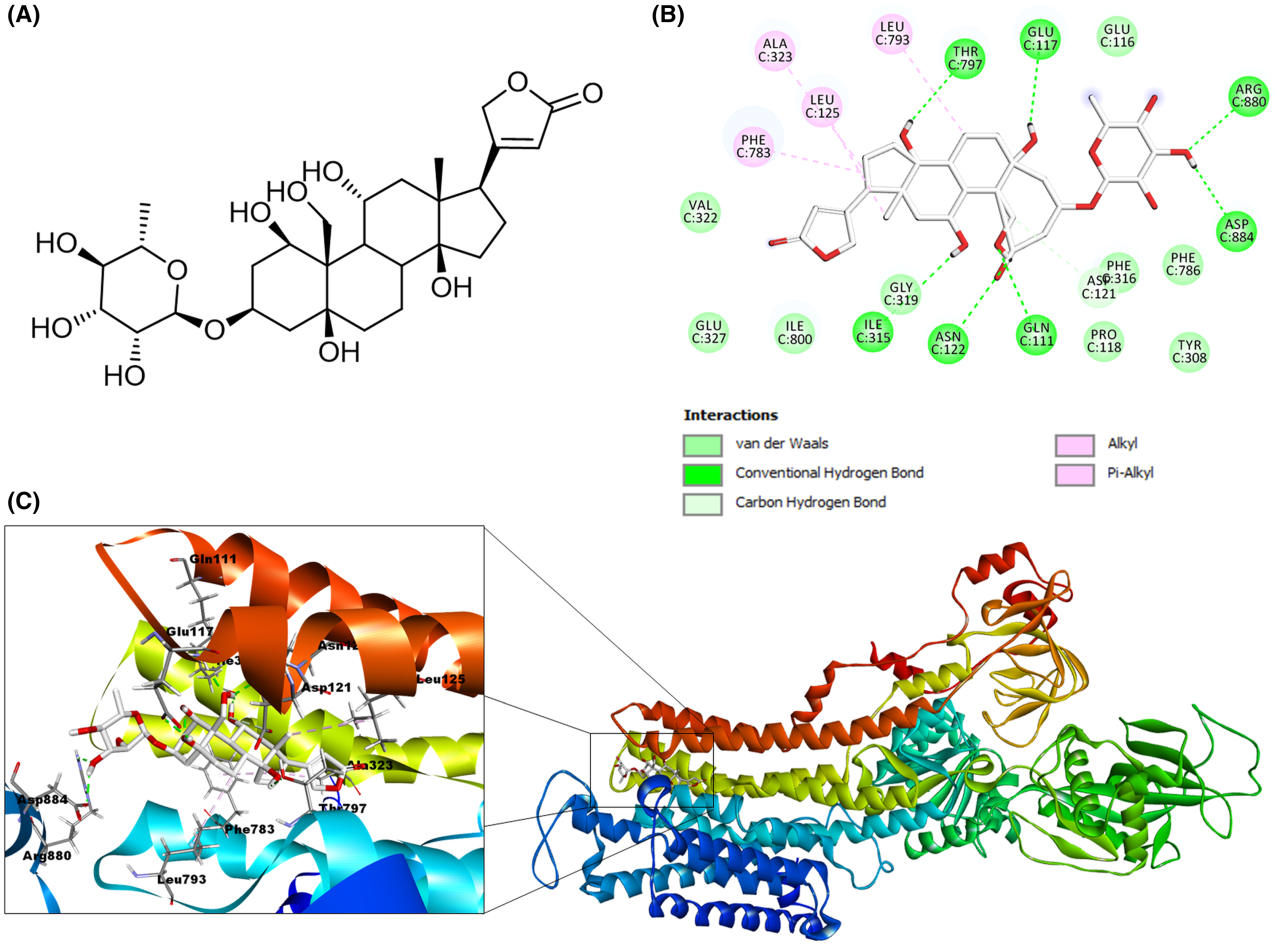
Molecular docking of reference ligand used in molecular modeling with the receptor Na^+^/K^+^-ATPase (PDB 4HYT). (A) Structure of ouabain. (B) 2D interaction map of reference ligand ouabain (white) with the receptor Na^+^/K^+^-ATPase. (C) Molecular modeling of reference ligand ouabain with the receptor Na^+^/K^+^-ATPase. A green dash line marked hydrogen bonds. A pink dash line marked hydrophobic interactions.

Molecular docking was used to predict the binding modes of Na^+/^K^+^-ATPase receptor-CTS ligand complexes using MOE software. The intramolecular contacts in the docked poses of cardenolides ligands bound to Na^+^/K^+^-ATPase were identified using the Discovery Studio Visualizer. To validate the molecular docking process, we carried out a re-docking of co-crystallized ligand ouabain into the binding site of Na^+^/K^+^-ATPase. The re-docked ouabain results are presented in Fig. 6. The re-docked ouabain (white) showed an RMSD value of 1.067 Å and binding energy of −9.6539 kcal/mol. As shown on the 2D interactive map (Fig. 6B), the validation model of the reference ligand presented hydrophobic interactions and hydrogen bonds similar to the crystallographic model. In the case of hydrogen bonds, we can highlight the interaction of residue Thr^797^ with the β-hydroxyl group at position 14, the bonds among amino acid Gln^111^ and the β-hydroxyl group at position 19, and a β-hydroxyl group at position 1, the hydrogen bond by residue Glu^117^ and the β-hydroxyl group at position 5, and the interaction between the amino acid Asn^122^ with the β-hydroxyl group at position 1. In addition, this validation model presented other hydrogen bonds with different amino acid residues as Ile^315^ with the β-hydroxyl group at position 11. The Asp^884^ and Arg^880^ with the β-hydroxyl group at position 3′ of the sugar moiety. Furthermore, the validation model reveals diverse hydrophobic interactions, the most remarkable are the alkyl interactions of Phe^783^ with the B and D steroidal rings, the Leu^125^ with the methyl group at position 18, the Ala^323^ residue with the D steroidal ring, and the amino acid Phe^316^ and the methyl group at position 6′ of the sugar moiety. Considering that the hydrophobic interactions and the crystallographic structure were the same and that most hydrogen bonds were similar to 4HYT protein, we conclude that the model validation and conditions of molecular docking were acceptable in our work.

### Molecular docking of cardenolides isolated from *A. subulata* with the Na^+^/K^+^-ATPase

**Figure 7:**
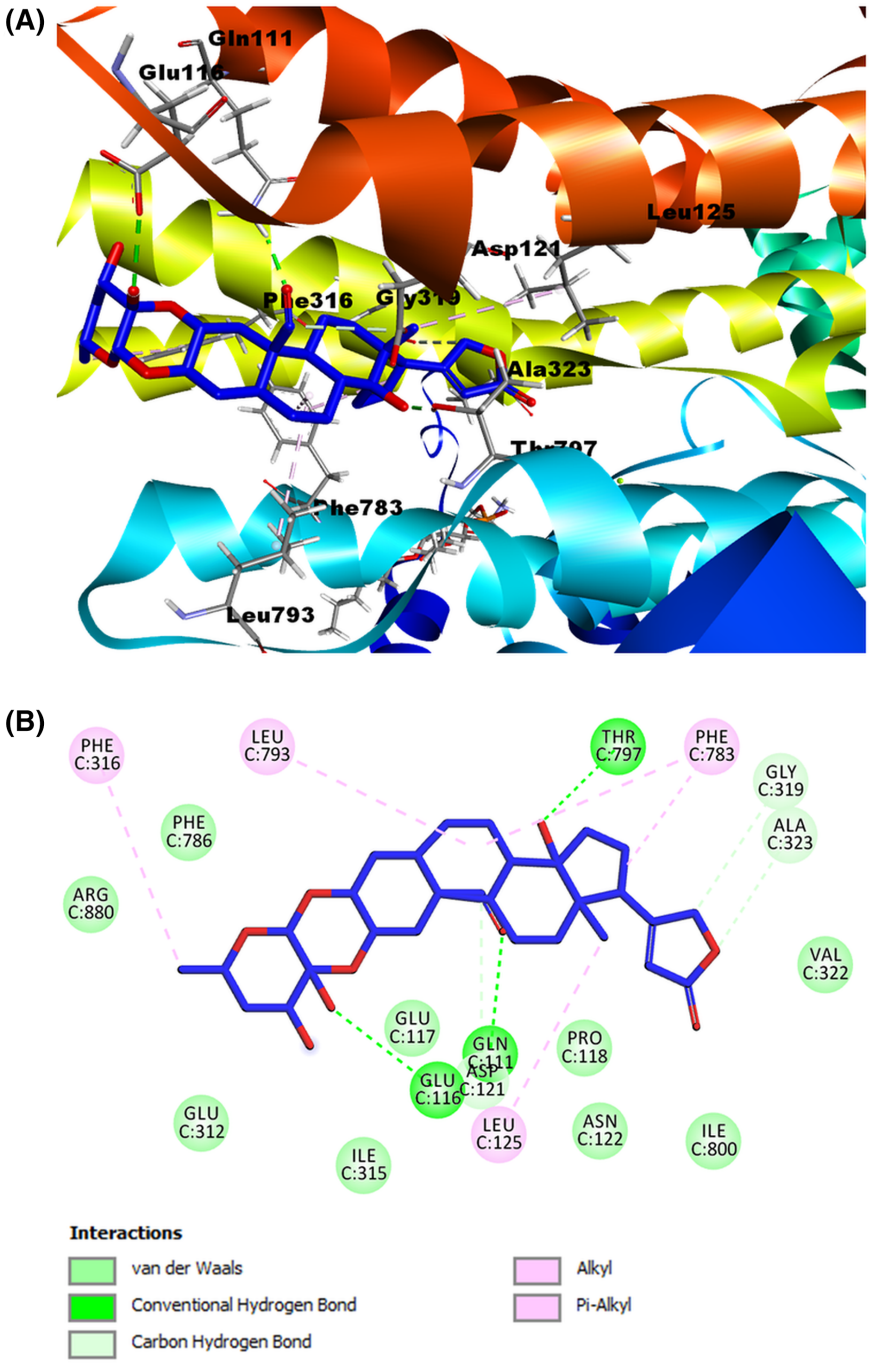
Molecular docking of Na^+^/K^+^-ATPase and the ligand calotropin (blue). (A) Molecular modeling of cardenolide calotropin and the Na^+^/K^+^-ATPase. (B) 2D interaction map of cardenolide calotropin as a ligand with the Na^+^/K^+^-ATPase as the receptor. A green dash line marked hydrogen bonds. A pink dash line marked hydrophobic interactions.

After the validation stage, we performed the molecular docking of cardenolides selected as ligands. We generated 23 possible receptor conformations with the calotropin ligand (shown as the blue molecule in Fig. 7A). Pose number 6 was selected based on the best RMSD value (1.05 Å) and binding energy (−7.2007 kcal/mol). As indicated in the 2D interactive map (Fig. 7B), the ligand calotropin can interact with the receptor due to several hydrogen bonds, highlighting the interaction among the amino acid residue Thr^797^ and the β-hydroxyl group at position 14 of cardenolide. In addition, the interaction between the residue Gln^111^ and the aldehyde group at position 19 is present in the crystallographic structure of Na^+^/K^+^-ATPase with the reference cardenolide ouabain. It is also important to mention the hydrogen bond between the β-hydroxyl group of position 3′ and the extracellular residue Gln^116^. This model also showed some hydrophobic interactions, the most remarkable between Phe^783^ residue with the B and D steroidal rings of the molecule, the pi-alkyl interaction among the Phe^786^ residue and the methyl group of position C6′, and the alkyl interaction of the amino acid Leu^125^ and the methyl group at position 18, all of these hydrophobic interactions were observed in the ouabain-Na^+^/K^+^-ATPase complex. This fact allows us to suppose that the cardenolide calotropin can establish a stable complex with the Na^+^/K^+^-ATPase.

**Figure 8:**
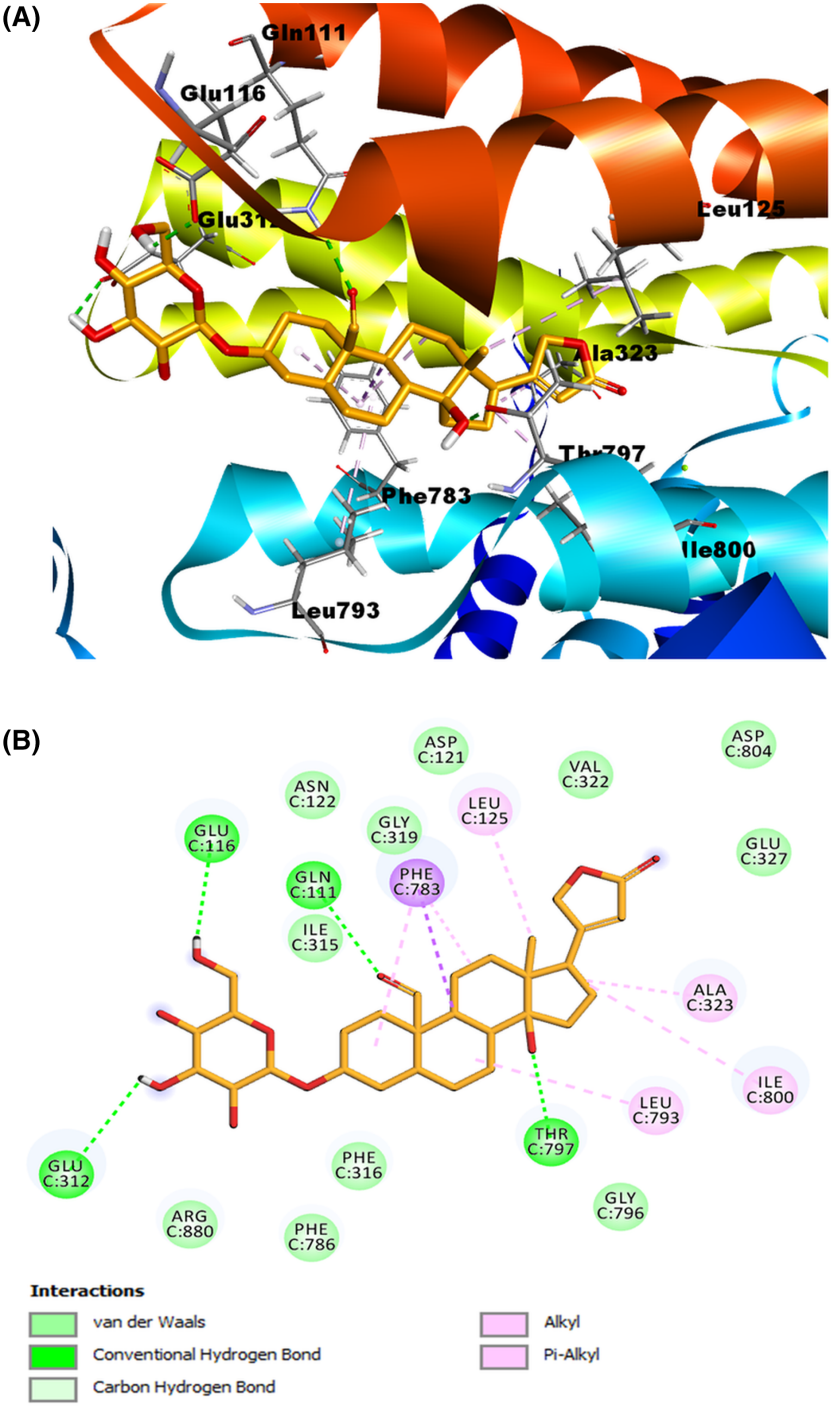
Molecular docking of Na^+^/K^+^-ATPase and the ligand C3OG (orange). (A) Molecular modeling of C3OG and the Na^+^/K^+^-ATPase. (B) 2D interaction map of C3OG as a ligand into the Na^+^/K^+^-ATPase as the receptor. Hydrogen bonds were represented as a green dash line. Hydrophobic interactions were marked by the pink and purple dash lines.

Meanwhile, for the ligand corotoxigenin 3-O-glucopyranoside (C3OG) (shown as the orange molecule in Fig. 8A), we obtained 27 possible conformations. For display purposes, we selected pose number 1, which had a lower RMSD value, and a more favorable binding energy, with 1.225 Å and −9.2133 Kcal/mol, respectively. As presented in (Fig. 8B), the 2D interactive map shows that this compound can interact with several amino acid residues of the Na^+^/K^+^-ATPase active site. The hydrogen bond interaction of Thr^797^ residue with the β-hydroxyl group at position 14, the bond between Gln^111^ amino acid and the aldehyde group at position 19, and the interactions of Glu^116^ and Glu^312^ residues with the hydroxyl groups at position 3′ and 6′ respectively were the main interactions in this model. According to the reference ligand, this docking could make three of the four hydrogen bonds of the ouabain-Na^+^/K^+^-ATPase complex possible. This is due to the structural similarity of the C3OG and the ouabain. The most remarkable bonds of the hydrophobic interactions are the pi-sigma interaction by Phe^783^ residue with the steroidal ring B and the pi-alkyl bond with the steroidal ring A and C. Other alkyl interactions to stand out are the ones between the Leu^125^ residue with the methyl group at position 18, the interaction of steroidal ring B with the Leu^793^ amino acid, and the bonds of Ala^323^ and Ile^800^ with the cyclopentane structure (steroidal ring D). Except for the interaction among Ile^800^, all the hydrophobic interactions match the interactions of the crystallographic data and our validation model with the reference ligand, which increases the possibility that this cardenolide can form a stable ligand-receptor complex.

As shown in the summary results in (Table 3), both cardenolides used as ligands in our models and the reference ligand ouabain presented almost the same interactions. We found the most remarkable interaction was the alkyl and Phe^783^ interaction, shown in all models, including the validation models. Additionally, the Ala^323^ residue also formed interactions with the ligands, either as a non-conventional hydrogen bond with the lactone ring or as an alkyl interaction with the steroidal ring D. Both amino acids seem to influence the conformation and stabilization of the molecules on the binding site. Another interaction to highlight is the alkyl interaction with the Leu^125^ residue and the methyl group at position 18, presented in all the ligands used. The hydrogen bonds showed similar behavior, especially with the interaction among Thr^797^ and the β-hydroxyl groups. This interaction was found in all the models. The hydrogen bond between Gln^111^ and the substituent group at position 19, indicated that these aldehyde or hydroxyl substitutions influence the stability of the cardenolide-protein complex.

**Table 3:** Data of binding energy, RMSD calculations, number of hydrogen bonds, and contacting receptor residues of ligands using molecular docking.

Ligand	Binding energy (Kcal/mol)	RMSD (Å)	Hydrogen bonds	Contacting receptor residues
Ouabain	−9.6539	1.067	7	Gln111, Glu116, Asp121, Asn122, Leu125, Ile315, Ala323, Phe783, Leu793, Thr797, Arg880, Asp886
Calotropin	−7.2007	1.053	3	Gln111, Glu116, Asp121, Leu125, Gly319, Ala323, Phe783, Leu793, Thr797
Corotoxigenin 3-O-glucopyranoside	−9.2163	1.225	4	Gln111, Glu116, Leu125, Glu312, Ala323, Leu793, Phe783, Thr797, Ile800

**Note:**

The values of RMSD are expressed in angstroms.

## Discussion

Cardiotonic steroids are potent inhibitors of the Na^+^/K^+^-ATPase and have been used to treat congestive cardiac affections for a hundred years (Reinhard et al., 2013). In this work, we demonstrated the capacity of calotropin and corotoxigenin 3-O-glucopyranoside (C3OG) isolated from *A. subulata* to inhibit the enzymatic activity of Na^+^/K^+^-ATPase by *in vitro* assays, and we performed some molecular models to describe the possible way of this compounds interact with the Na^+^/K^+^-ATPase. The results have shown that calotropin and C3OG inhibited the enzymatic activity of Na^+^/K^+^-ATPase at concentrations below 1.0 μM. Furthermore, the inhibitory effect of these cardenolides is comparable to inhibitory activity presented by common cardenolides such as uscharin, frugoside, or desglucouzarin (Pederson et al., 2020). Also, we give the first report of C3OG and their inhibitory effect on Na^+^/K^+^-ATPase activity. C3OG, also called glucocorotoxigenin, is a cardenolide identified from *Coronilla scorpioides*, and there are a few reports about their biological activities (Rascón-Valenzuela et al., 2015; Al-Snafi, 2016).

The IC_50_ value of calotropin obtained in the present work was consistent with the literature reports. According to authors such as Agrawal et al. (2021), calotropin had an IC_50_ value of 0.27 μM on porcine cerebral cortex Na^+^/K^+^-ATPase activity. Other reports about the inhibitory effect on Na^+^/K^+^-ATPase by cardenolides as calotoxin or frugoside presented similar IC_50_ values to those observed in calotropin (Pederson et al., 2020). Studies in cardenolides like digoxin, used as a reference inhibitor, showed an IC_50_ value of 0.23 μM on Na^+^/K^+^-ATPase activity (Ren et al., 2020). Considering this evidence is remarkable the great inhibitory potential of calotropin on Na^+^/K^+^-ATPase.

Comparing the inhibitory effect of cardenolides isolated from *A. subulata* with ouabain, it is evident that IC_50_ values of calotropin and ouabain are very close (Table 1). However, a significant difference exists between the inhibitory effect of C3OG with calotropin and ouabain. This inhibition difference is also appreciable in the K_i_ values because C3OG presented a higher K_i_ value than calotropin and ouabain, which implies that C3OG has less affinity to Na^+^/K^+^-ATPase than the other evaluated compounds.

Furthermore, the kinetic analysis showed that calotropin and C3OG exhibited an uncompetitive inhibition of Na^+^/K^+^-ATPase activity. This kind of inhibition has been reported in compounds such as digitoxin, gitoxin, strebloside, and glucostrebloside (Krstic et al., 2004; Bai et al., 2019). The uncompetitive inhibitors can bind only to the enzyme-substrate complex and not the free enzyme. Also, the substrate-binding may cause conformational changes in the enzyme (Palmer & Bonner, 2011). Therefore, the potency of uncompetitive inhibitors is increased with greater substrate concentrations and inhibition. Also, uncompetitive inhibition provides better odds to maximize the strength and specificity (Boulton et al., 2018). This inhibition is supported by the way ouabain binds to the Na^+^/K^+^-ATPase. It has been established in numerous studies that ouabain inhibits the Na^+^/K^+^-ATPase by binding in a specific pocket that is accessed by the extracellular side of the enzyme. The Na^+^/K^+^-ATPase alternates among two conformational states during the catalytic cycle (E_1_–E_2_). Therefore, ouabain can bind to the enzyme’s phosphorylated E_2_ form (E_2_P) with great affinity (Qiu et al., 2005). This fact supports crystallographic studies with ouabain and the Na^+^/K^+^-ATPase because, in these studies, the binding site of this molecule does not modify the phosphorylation site of ATP. Instead, it stabilizes the enzyme in an E_2_P conformation. In addition, inside the binding site of ouabain to Na^+^/K^+^-ATPase, the cardenolide can establish interactions with amino acid residues that are considered essential to increase the binding affinity (Laursen et al., 2013).

The importance of amino acid residues on the active site of Na^+^/K^+^-ATPase has been widely studied. In nature, insects express a natural immunity against cardenolides due to modification in some amino acid residues on the structural protein. Studies with Na^+^/K^+^-ATPase isolated from monarch butterflies have demonstrated that cardenolides as ouabain possess lower binding affinity due to the change of amino acid Asn^122^ by His^122^ (Petschenka et al., 2018). Other studies with the Na^+^/K^+^-ATPase purified from *Oncopeltus fasciatus* and ouabain showed the modification of amino acids as Gln^111^-Thr^111^, Asn^122^-His^122^, Phe^786^-Asp^786^, and Thr^797^-Ala^797^ decrease the binding affinity of the cardenolide to the enzyme (Bramer et al., 2015). Mutagenic studies with Na^+^/K^+^-ATPase and H^+^/K^+^-ATPase demonstrated amino acid residues as Glu^312^, Val^314^, Ile^315^, Gly^319^, Phe^783^, Thr^797^, and Asp^804^ are significant to ouabain interaction with the Na^+^/K^+^-ATPase (Yan-Qiu et al., 2003; Qiu et al., 2005). Furthermore, it is known that cardenolides present lower toxicity on murine Na^+^/K^+^-ATPase due to the modification of amino acid residues such as Gln^111^-Arg^111^ and Asn^122^-Asp^122^ (Akimova et al., 2015). This fact highlights the importance of conserved residues such as Phe^786^, Thr^797^, Gln^111^, or Asp^121^ in the Na^+^/K^+^-ATPase for cardenolides; they allow the compounds to bind adequately to the protein and trigger the inhibitory effect.

According to our observations in molecular modeling, calotropin and C3OG present hydrogen bond interactions with the amino acid residue Thr^797^ and the hydroxyl group at position 14 and Gln^111^ residue with the oxygen from the aldehyde group at position 19. In both compounds, the oxygen of aldehyde and hydroxyl group at position 14 act as hydrogen bond acceptors, similar to the hydroxyl group at the same position in the ouabain-Na^+^/K^+^-ATPase complex. In addition, the hydrogen bonds formed with functional groups of the sugar moiety of cardenolides and amino acid residues were more diverse, and almost all hydroxyl groups of sugar moiety act as hydrogen bond donors. According to Laursen et al. (2013), the sugar moiety of ouabain is exposed to the extracellular environment in the cavity, where amino acid with polar residues, like Glu^116^, Glu^312^, Arg^880^, and Asp^884^, increases the possibility of interactions between the functional groups of sugar moiety and these amino acid residues. Additionally, it has been demonstrated that one of the functions of sugar moiety in ouabain is to stabilize the CTS-Na^+^/K^+^-ATPase complex through the interaction of hydroxyl group on the sugar moiety, which work as hydrogen bond donors or acceptors. This fact depended on the orientation, position, and amount of hydroxyl groups of the sugar moiety (Cornelius, Kanai & Toyoshima, 2013). According to studies of CTS on Na^+^/K^+^-ATPase activity, CTS that presents a sugar moiety is regularly better Na^+^/K^+^-ATPase inhibitors than the corresponding aglycones (Iyer et al., 2010; Azalim et al., 2020). This fact is observable in studies with sugar moiety on digitoxin O-glycosides analogs and digitoxin MeON-glycosides analogs, where digitoxin O-glycosides analogs showed better antiproliferative and apoptotic effects than digitoxin MeON-glycosides and the aglycone digitoxigenin (Wang et al., 2010; Wang et al., 2011a). Also, studies of digitoxin analogs with different sugar moieties showed that the carbohydrate-binding site on the target could take distinct binding orientations simultaneously (Wang, Rojanasakul & O’Doherty, 2011b). These facts explain the different amino acid residues that can form interactions with the sugar moiety of the cardenolides used as ligands in the molecular models.

On the other hand, cardenolides calotropin and C3OG formed one or more hydrophobic interactions with some of the amino acids reported on the crystallographic structure with the Na^+^/K^+^-ATPase, highlighting the bonds with Leu^125^, Ala^323^, Leu^793^, and Phe^783^. These interactions were presented mostly by steroidal rings interactions with Phe^783^ and Leu^793^ residues or the Leu^125^ with the methyl group at position 18. In the ouabain-Na^+^/K^+^-ATPase complex, the hydrophobic interactions presumably execute a stabilizing function of the steroidal core due to the interactions made by phenylalanine residues on the binding site with steroidal rings of cardenolide (Ogawa et al., 2009; Yatime et al., 2009). Moreover, the calotropin presented carbon-hydrogen bonds with Ala^323^, and Gly^319^ residues to the lactone ring, specifically with oxygen and hydrogens at position 22. Comparing the affinity potency of cardenolides evaluated on Na^+^/K^+^-ATPase activity and the molecular modeling in the present work is possible to infer that the difference in the K_i_ and IC_50_ values obtained during the inhibitory assays has a relation with the capacity of calotropin and C3OG to interact and bind with the enzyme and form a stable complex. The lack of interactions with amino acids such as Asp^122^, Gly^319^, and Ala^323^ considered essential to cardenolides to bind to Na^+^/K^+^-ATPase can cause a decrease in the affinity binding of C3OG in comparison to calotropin that presented more interactions like the reference ouabain on the binding site, reflected on the values of K_i_ and IC_50_ shown by this compound. Conforming to the experimental evidence obtained in the present work is demonstrated that cardenolides isolated from *A. subulata* can inhibit the Na^+^/K^+^-ATPase with the same type of inhibition. Moreover, both cardenolides evaluated can take a conformation like ouabain into the binding site on the enzyme as shown in the molecular modeling, and the difference in the interactions of cardenolides and the amino acid residues influence the effect of the compounds on the Na^+^/K^+^-ATPase activity.

This fact can be extrapolated to other biological effects of these cardenolides as cytotoxicity and structure-activity relationship studies. For example, cytotoxicity studies carried out by Rascón-Valenzuela et al. (2015) with calotropin and C3OG against human cancer cell lines as A549, PC-3, and LS-180 showed that calotropin was more active in all cell lines evaluated than C3OG with IC_50_ values in nanomolar order to calotropin and micromolar order to C3OG. Also, studies of QSAR realized with *double linked-type* cardenolides showed that physicochemical properties as hydrogen bonds acceptors and hydrophobic features are involved in the cytotoxic effect of these cardenolides against the A549 cell line (Meneses-Sagrero et al., 2021). This reinforces the information provided by molecular modeling and the inhibition assays of the present work and suggests that the cytotoxic activity of calotropin and C3OG are related to the inhibition of the Na^+^/K^+^-ATPase.

Furthermore, numerous studies with cardenolides have demonstrated apoptosis induction and cancer cell growth inhibition by binding to the Na^+^/K^+^-ATPase pump. Digitoxin-bound to Na^+^/K^+^-ATPase activates Src, PI3K, PLC, and Ras/MAPK pathways, leading to antiproliferative downstream effects related to cell growth, apoptosis, and subsequent cell cycle arrest caused by an increased level of p21^Cip1^ (Langenhan et al., 2005). Also, this cardenolide increases the caspase 3, 8, 9, and cytochrome C expression on lung cancer cell lines (Elbaz et al., 2012). According to Mo et al. (2016), calotropin activates the mitochondrial apoptotic pathway, accompanied by an increase in Bax/Bcl-2 ratio, decreased mitochondrial membrane potential, and increased reactive oxygen species (ROS) production and activation of caspases 3 and 9. Rascón-Valenzuela et al. (2016) reported that calotropin and C3OG induce apoptosis by caspases 3, 8, and 9 activating the A549 cell line. Malayoside is a cardenolide with a similar structure to C3OG, which showed a cytotoxic and apoptotic effect on the NCI-H460 lung cell line. These effects were associated with the expression of Nur77, activated by p38 and the ERK1/2 signaling pathway (Hu et al., 2021). The inhibition of Na^+^/K^+^-ATPase by calotropin and C3OG causes the activation of the Src and MAPK-ERK1/2 signaling pathway to promote the expression of Nur77 and their translocation to mitochondrial. This translocation can activate the cytochrome C release to the cytoplasm and activate the caspases 9 and 3 to induce apoptosis in cells. Nevertheless, it is crucial to continue the evaluations with calotropin and C3OG to determine the actual signaling pathways involved in their mechanism of action and their possible association with Na^+^/K^+^-ATPase inhibition.

## Conclusions

The results of the Na^+^/K^+^-ATPase inhibition assays by the cardenolides isolated from *A. subulata* demonstrated the ability of calotropin and C3OG to inhibit the enzyme’s enzymatic activity by an uncompetitive enzymatic mechanism. This observation supported the molecular models because both compounds have shown interactions with amino acid residues considered essential for a good binding with the protein complex, similar to the reference ouabain. Furthermore, these findings reinforce the concept that cardenolides calotropin and C3OG likely cause their cytotoxic effects through the inhibition of Na^+^-K^+^-ATPase.

## Supplemental Information

10.7717/peerj.13524/supp-1 Supplemental Information 1 Kinetic analysis of Na+/K+-ATPase activity. Kinetic analysis of Na+/K+-ATPase activity from the porcine cerebral cortex in the absence or presence of cardenolide ouabain at different concentrations

10.7717/peerj.13524/supp-2 Supplemental Information 2 Inhibitory effect of ouabain on Nat/K*-ATPase activity. Inhibitory effect of ouabain on Nat/K*-ATPase activity. The results are the three independent experiments y concentration by triplicate. The solid lines represent the theoretical curves according to the non-lineal regression fitting.

10.7717/peerj.13524/supp-3 Supplemental Information 3 Inhibition of Nat/K*-ATPase from the porcine cerebral cortex in presence or absence of 0.1 and 1.0 uM of ouabain at different concentrations of substrate. Inhibition of Nat/K*-ATPase from the porcine cerebral cortex in presence or absence of 0.1 and 1.0 uM of ouabain at different concentrations of substrate. A) Michaelis-Menten fitting of ouabain at 0.1 (M (blue squares), 1.0 uM (blue triangles), and without inhibitor (blue circles). B) Lineweaver-Burk plot of ouabain at 0.1 uM (blue squares), 1.0 mM (blue triangles), and without inhibitor (blue circles). Data were determined by three independent assays, each one with 3 replicates by evaluated concentration.

10.7717/peerj.13524/supp-4 Supplemental Information 4 Raw data.

10.7717/peerj.13524/supp-5 Supplemental Information 5 The results of the analysis.

10.7717/peerj.13524/supp-6 Supplemental Information 6 Ouabain Molecular Docking Data.

10.7717/peerj.13524/supp-7 Supplemental Information 7 Calotropin Molecular Docking Data.

10.7717/peerj.13524/supp-8 Supplemental Information 8 Corotoxigenin 3-O-glucopyranoside Molecular Docking Data.

## Additional Information and Declarations

### Data Availability

The following information was supplied regarding data availability: The raw data are available in the Supplemental Files.
