# Supplementary material for: Calotropin and corotoxigenin 3-O-glucopyranoside from the desert milkweed Asclepias subulata inhibit the Na+/K+-ATPase activity
Source: PeerJ. 2022 Jun 2;10:e13524. doi: 10.7717/peerj.13524 (PMC9167584; doi:10.7717/peerj.13524)
Supplement: Supplemental Information 1 — Kinetic analysis of Na+/K+-ATPase activity from the porcine cerebral cortex in the absence or presence of cardenolide ouabain at different concentrations [file peerj-10-13524-s001.pdf]

Table SI. Kinetic analysis of Na^+/K^+-ATPase activity from the porcine cerebral cortex in the absence or presence of cardenolide ouabain at different concentrations.

Compound	Concentration (μM)	K_m (mM)	V_{max} (μmolPi/mg/min)	K_i (μM)	Type of inhibition
Ouabain	0.0	0.88 ± 0.09	3.8 ± 0.6	0.16 ± 0.04	Uncompetitive
	0.1	0.47 ± 0.06	2.11 ± 0.14		
	1.0	0.12 ± 0.01	0.66 ± 0.04		

All values were obtained from triplicate independent assays. The data are expressed as mean ± standard deviation. The values of K_m and V_{max} were calculated by data fitting Michaelis-Menten non-linear regression. K_m is expressed in mM. V_{max} is expressed as μmol of Pi released/mg of protein/min.
