# Supplementary figures and images for: Calotropin and corotoxigenin 3-O-glucopyranoside from the desert milkweed Asclepias subulata inhibit the Na+/K+-ATPase activity
Source: PeerJ. 2022 Jun 2;10:e13524. doi: 10.7717/peerj.13524 (PMC9167584; doi:10.7717/peerj.13524)

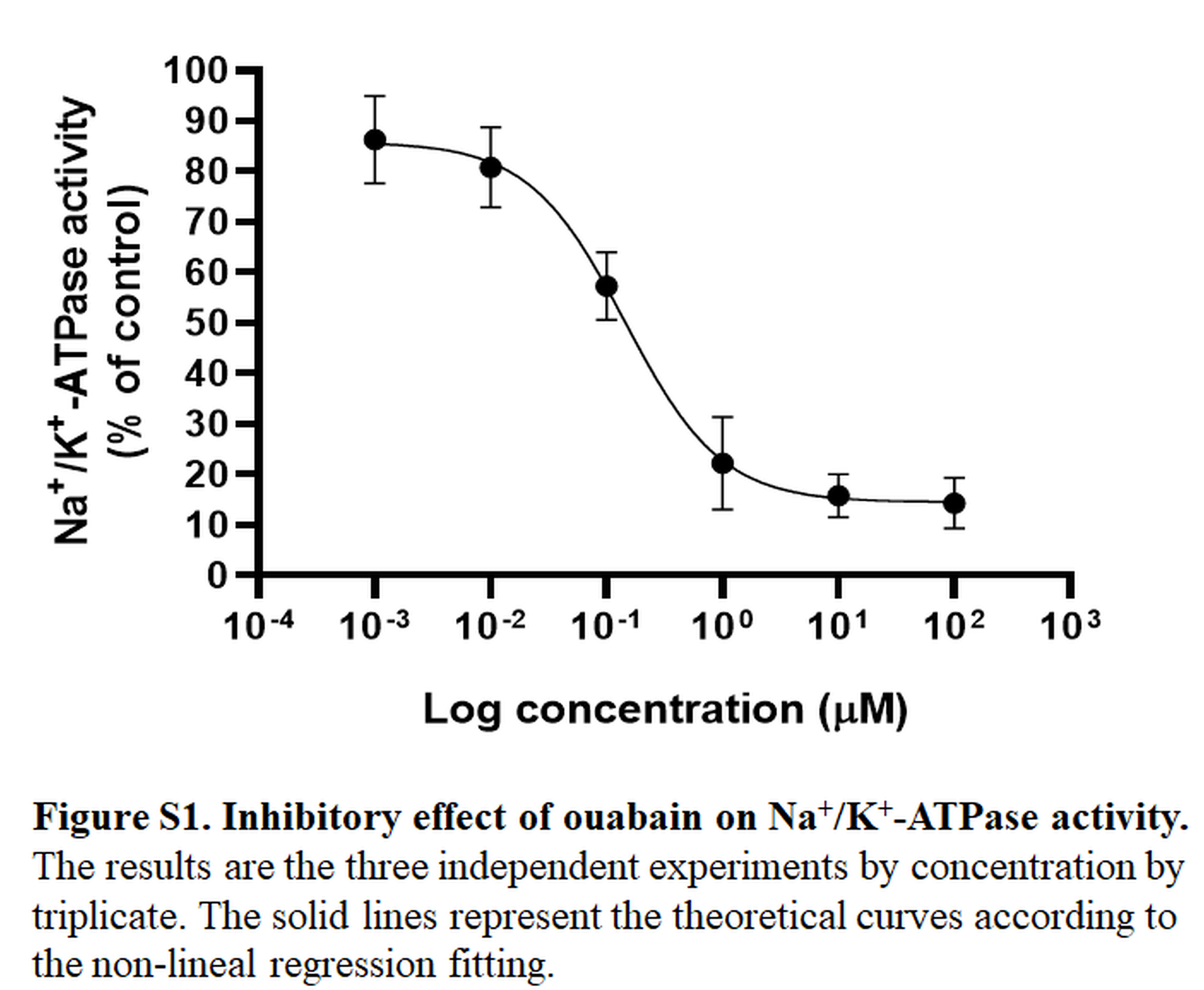

Supplement: Supplemental Information 2 — Inhibitory effect of ouabain on Nat/K*-ATPase activity. The results are the three independent experiments y concentration by triplicate. The solid lines represent the theoretical curves according to the non-lineal regression fitting. [file peerj-10-13524-s002.png]

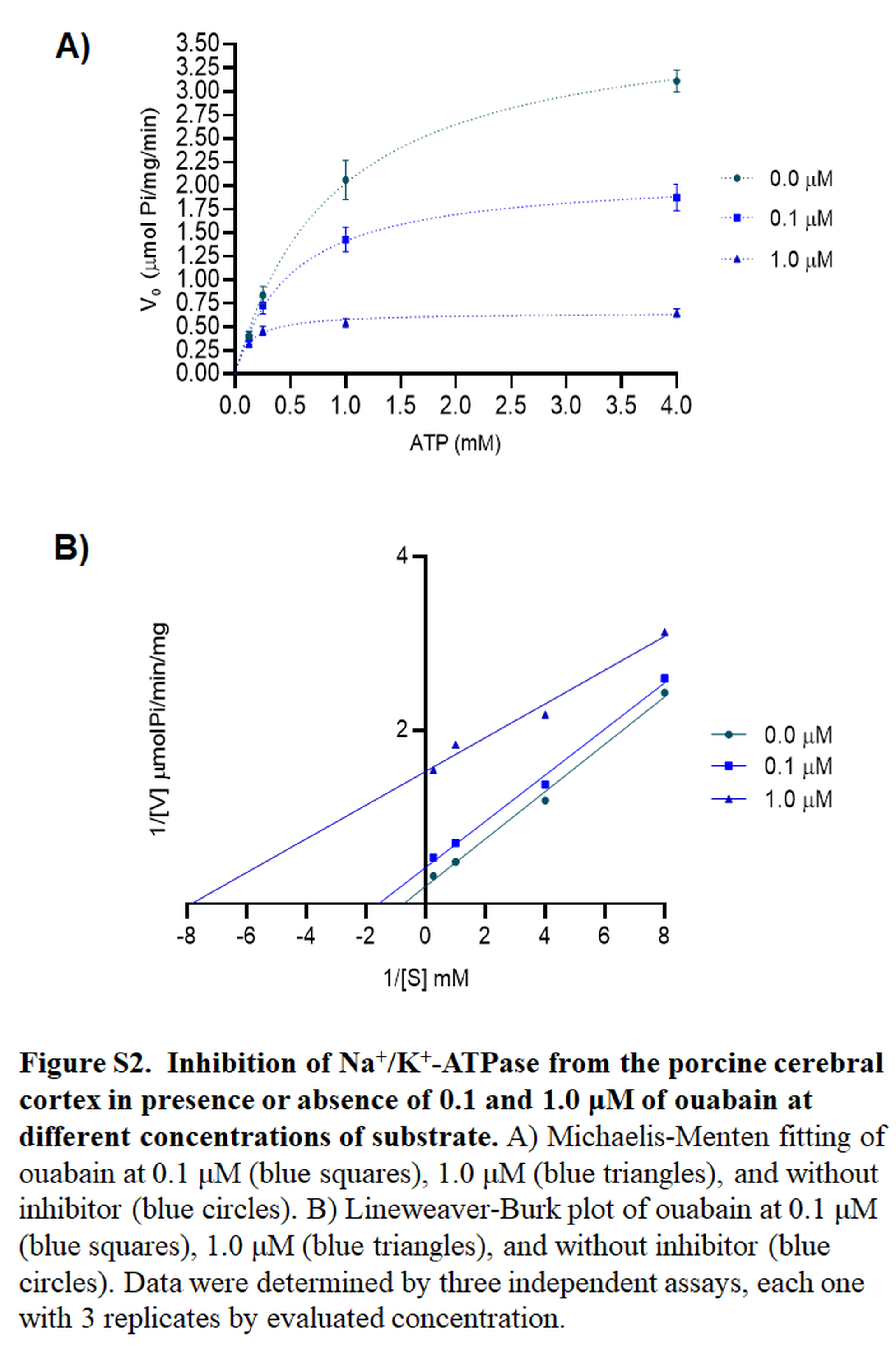

Supplement: Supplemental Information 3 — Inhibition of Nat/K*-ATPase from the porcine cerebral cortex in presence or absence of 0.1 and 1.0 uM of ouabain at different concentrations of substrate. A) Michaelis-Menten fitting of ouabain at 0.1 (M (blue squares), 1.0 uM (blue triangles), and without inhibitor (blue circles). B) Lineweaver-Burk plot of ouabain at 0.1 uM (blue squares), 1.0 mM (blue triangles), and without inhibitor (blue circles). Data were determined by three independent assays, each one with 3 replicates by evaluated concentration. [file peerj-10-13524-s003.png]
